# Association of Social Engagement with Brain Volumes Assessed by Structural MRI

**DOI:** 10.1155/2012/512714

**Published:** 2012-09-11

**Authors:** Bryan D. James, Thomas A. Glass, Brian Caffo, Jennifer F. Bobb, Christos Davatzikos, David Yousem, Brian S. Schwartz

**Affiliations:** ^1^Rush Alzheimer's Disease Center, Department of Internal Medicine, Rush University Medical Center, Chicago, IL 60612, USA; ^2^Department of Epidemiology, Johns Hopkins Bloomberg School of Public Health, Baltimore, MD 21205, USA; ^3^Department of Biostatistics, Johns Hopkins Bloomberg School of Public Health, Baltimore, MD 21205, USA; ^4^Department of Radiology, University of Pennsylvania School of Medicine, Philadelphia, PA 19104, USA; ^5^Department of Radiology, Johns Hopkins School of Medicine, Baltimore, MD 21205, USA; ^6^Department of Environmental Health Sciences, Johns Hopkins Bloomberg School of Public Health, Baltimore, MD 21205, USA; ^7^Department of Medicine, Johns Hopkins School of Medicine, Baltimore, MD 21205, USA

## Abstract

We tested the hypothesis that social engagement is associated with larger brain volumes in a cohort study of 348 older male former lead manufacturing workers (*n* = 305) and population-based controls (*n* = 43), age 48 to 82. Social engagement was measured using a summary scale derived from confirmatory factor analysis. The volumes of 20 regions of interest (ROIs), including total brain, total gray matter (GM), total white matter (WM), each of the four lobar GM and WM, and 9 smaller structures were derived from T1-weighted structural magnetic resonance images. Linear regression models adjusted for age, education, race/ethnicity, intracranial volume, hypertension, diabetes, and control (versus lead worker) status. Higher social engagement was associated with larger total brain and GM volumes, specifically temporal and occipital GM, but was not associated with WM volumes except for corpus callosum. A voxel-wise analysis supported an association in temporal lobe GM. Using longitudinal data to discern temporal relations, change in ROI volumes over five years showed null associations with current social engagement. Findings are consistent with the hypothesis that social engagement preserves brain tissue, and not consistent with the alternate hypothesis that persons with smaller or shrinking volumes become less socially engaged, though this scenario cannot be ruled out.

## 1. Introduction

Social engagement, the performance of meaningful social roles for either leisure or productive activity, has been shown to be associated with better cognitive function and lowered rates of cognitive decline and dementia in older adults [1–4]. Yet many questions remain regarding how social engagement can potentially get “under the skull” to preserve cognitive abilities. Inconsistencies in measurement across studies is frequent with a number of overlapping constructs such as social activity [1, 5], social networks [6, 7], and social support [8] linked to cognitive outcomes; each has been theorized to affect the brain through separate mechanisms. Yet the neurological mechanisms that could lead to preservation of cognitive function remain unclear and perhaps the largest obstacle is a lack of research to directly explore the biological effects of social engagement on the brain. A popular hypothesis is that social engagement helps to build a brain reserve capacity that allows the brain to tolerate neuropathologic damage due to aging or disease without deterioration of cognitive abilities [9, 10]. In a case of “use it or lose it,” remaining socially engaged as one ages may build this brain reserve through neuroplastic changes in the brain such as attenuated neuronal loss, or increased synaptic count [11–13] or the growth of new neurons [14]—all of which could be reflected in an increase or attenuated shrinking of brain volume. In the context of aging, larger brain volumes are associated with better cognitive function [15, 16], and preservation of cognitive function in the face of neuropathology [17]. Demonstrating a link between social engagement and larger brain volumes would provide support for the brain reserve hypothesis and our understanding of the neurological mechanisms at play.

We examined the relationship between social engagement and brain volumes using two complementary methods, a region-of-interest (ROI) analysis to investigate recognized anatomical brain regions, and voxel-based morphometry (VBM) to explore unbiased associations across the entire brain. Utilizing available longitudinal MRI data, we were also able to evaluate whether change in ROI volumes over five years prior to assessment of social engagement was associated with current level of social engagement in order to better discern temporal relations. We hypothesized that more socially engaged persons have larger brain volumes, especially for GM, which was found to evidence larger age-related declines in volume compared to WM in this population [18]. 

## 2. Methods

### 2.1. Study Population and Design

We used data from a study of lead exposure and cognitive function in former employees of a chemical manufacturing plant in the eastern United States and population-based controls with no history of occupational lead exposure [19]. The controls were selected from the same geographical residential as the former lead workers resided in using random selection from a telephone database and frequency-matched to lead workers for age, education, and race [20]. We used data from the third phase of this study when assessment of social engagement and a second MRI were obtained from study participants; an initial baseline structural MRI was acquired in phase 2, on average 5 years earlier. Detailed methods for study design and recruitment in phases 1 (1994–1997; 703 former lead workers and 130 controls, mean age 56 years) [20, 21] and 2 (2001–2003; 589 of 979 former lead workers and 67 of 131 controls completed MRI; mean age 56 at enrollment) [22] are described elsewhere. During phase 3 (2005–2008), 396 participants returned for an additional study visit. All phases of the study were reviewed and approved by the Johns Hopkins Bloomberg School of Public Health Committee on Human Research and written informed consent was obtained from all participants.

During phase 3, participants who completed the first MRI in phase 2 (589 former lead workers and 67 controls) were invited for a second MRI; 317 (54%) former lead workers and 45 (67%) controls completed a second MRI. Thus, two MRIs were obtained from 362 participants, representing 91% of the 396 participants who returned for phase 3 of the study. Nine of these had poor quality scans, leaving 353 participants with useable MRIs. Participants with phase 3 MRIs were on average younger than participants with no MRI or only a phase 2 MRI [18]. Five participants had missing data on social engagement; our final analysis included 348 participants.

### 2.2. Structural Magnetic Resonance Imaging Acquisition

A 3 T General Electric scanner was utilized for the phase 3 MRI. T1-weighted images were acquired using spoiled gradient recalled acquisition (SPGR) in steady state sequence (repetition time (TR) = 21 ms, echo time (TE) = 8 ms, field of view (FOV) = 24 cm, flip angle = 30° one excitation, voxel size = 0.9375 mm by 0.9375 mm by 1.5 mm, field of view 24 cm, matrix size 256 × 256). Methods for phase 2 MRI have been previously published [22].

### 2.3. Image Analysis

Quantitative analysis of MR volumes was completed using previously published methods [23]. First, extracranial tissue and brainstem structures were stripped. A validated specialized image analysis method was employed to segment the images into GM and WM. The CLASSIC algorithm [24] employs a 4-dimensional segmentation framework in which the first and second scans are considered jointly to minimize discrepancies between the two segmentations. The segmented images provide quantitative volumetric measures of total GM, WM, and brain (GM plus WM) matter.

To obtain volumes of predefined ROIs, regional analysis was performed via computerized template matching techniques previously reported and validated [22, 25]. In brief, a computerized image analysis algorithm based on pattern matching was used to warp a reference digital brain atlas to each participant's MRI. The resulting 20 nonmutually exclusive ROIs included the volumes of total brain, total GM, total WM, major lobar subdivisions, and a number of smaller structures. 

For the voxel-wise approach, regional analysis of volumes examined in normalized space (RAVENS) was used to yield brain maps for analysis of local volumetric differences not constrained by *a priori *anatomic definitions [23]. This method can provide confirmatory evidence of associations in predefined regions or provide additional insights into areas of the brain linked to social engagement that are not apparent from using the ROI approach. Using previously published methods [25], segmented images were transformed into a standard coordinate space using an elastic deformation algorithm. This procedure yields tissue density maps for GM and WM whose values are direct measurements of local tissue volumes. Associations of predictor variables with GM and WM volumes could then be examined on a voxel-by-voxel basis, not constrained by arbitrary anatomical boundaries, thereby revealing spatial patterns of such associations.

### 2.4. Social Engagement

Social engagement was measured for the first time in phase 3 of this study (i.e., at the time of the second MRI). The measure of social engagement came from the enacted function profile (EFP), a 20-item scale designed to measure multiple domains of enacted functional performance in older adults based on pre-existing theory regarding the measurement of actual functional performance (rather than theoretical functional capacity) in daily life [26]. The EFP asks respondents how often they have engaged in a number of common daily activities over the past week or month. To test our measurement theory and to correct for random measurement error, we used confirmatory factor analysis to examine the conditional independence of four domains of enacted function (social engagement, community involvement, self-care, and productive activities) and to derive factor scores to represent our theorized latent constructs. For the analysis, a factor-based score based on the 8 social engagement items was generated using MPLUS version 5. The social engagement items and scale details are included in the appendix.

### 2.5. Statistical Analysis

#### 2.5.1. ROI-Based Approach

We first evaluated whether higher social engagement was associated with larger volumes of predefined anatomical ROIs (dependent variable) in a cross-sectional analysis of phase 3 data. Associations between social engagement and brain volumes were examined using linear ordinary least squares regression modeling, with a separate model for each ROI. Because tibia lead is associated with smaller brain volumes [22], but was only available for lead workers, we first evaluated whether tibia lead level altered the association of interest or if it was appropriate to include both lead workers and controls in our main analyses without adjusting for lead. Tibia lead was not associated with social engagement and there was no evidence that the association of social engagement and brain volumes differed by control status or by tibia lead level, so we combined former lead workers and controls in all subsequent analyses. 

All models were adjusted for intracranial volume, handedness, and control status (versus former lead workers). Demographic and health factors that could confound the relationship between social engagement and brain volume included age (centered), race/ethnicity (all minorities versus whites), education (five categories, with high school plus trade school as the reference group), cardiovascular disease risk factors known to be associated with brain pathology (hypertension and diabetes), and tibia lead level (measured in lead workers only). Effect modification by age, education, race/ethnicity, cardiovascular risk factors, and control status was evaluated using models with cross-product terms. To facilitate comparisons across ROIs, standardized regression coefficients are presented. Model diagnostics were performed to examine model fit and influential points. Because the 20 ROIs are not independent, we did not adjust for multiple comparisons in this analysis choosing instead to report standard errors and unadjusted tests of associations. Analyses were performed with SAS 9.1 statistical software. 

#### 2.5.2. ROI-Based Analysis to Address Temporality

To discern temporal relations, we performed secondary analyses using the available longitudinal data from the first and second MRI. We modeled social engagement (measured at the time of the second MRI) as the *outcome variable* regressed upon the change in ROI volumes from first to second MRI (to address whether change in brain volumes over five years is associated with social engagement at the end of the interval). Standardized regression coefficients are presented. Strength of association and model fit for these models were compared to our main models.

#### 2.5.3. Voxel-Wise Approach

We next used voxel-based analysis to identify areas of GM and WM associated with social engagement. At each voxel we conducted linear regression of the voxel volume versus social engagement controlling for the aforementioned covariates. From the regression output we obtained a *t*-statistic for each voxel. We identified 3-dimensional clusters of 100 or more contiguous voxels exceeding the statistical threshold *t* >3.11 (corresponding to an uncorrected *P* value <0.001). To address multiplicity, we conducted a permutation test to assess the statistical significance of each cluster with respect to the permutation distribution of the largest cluster of suprathreshold *t*-statistics. More specifically, for 250 repetitions, we permuted the brain images (e.g., voxel volumes) across subjects, keeping the covariate data fixed. Then for each permuted dataset, we performed the same analysis as was done on the original dataset, identifying the largest cluster of contiguous voxels exceeding *t* >3.11. We finally obtained a *P* value for each cluster by calculating the proportion of repetitions for which the size of the cluster in question was greater than or equal to the largest cluster of the permuted data. This voxel-wise analysis was conducted separately for the gray and white matter maps.

## 3. Results

### 3.1. Descriptive Summary of Study Participants

Study participants were 48 to 82 years of age (mean (S.D.) = 65.2 (7.9)); the majority were white/non-Hispanic, had a high school education plus trade school, were hypertensive, and not diabetic (Table 1). The oldest individuals and the most educated were more socially engaged. Younger participants, white non-Hispanic persons, and those without hypertension or diabetes had larger brain volumes. There was a complex pattern of association between brain volumes and levels of education, as persons with a graduate degree had brain volumes similar to those with less than high school education; both groups had smaller total brain volumes compared with the high school plus trade school reference group. Participants in both the lowest and highest education groups were an average of 3.5 years older than participants in the middle categories. Former lead workers were less socially engaged than population-based controls but had larger brain volumes on average.

### 3.2. Associations of Social Engagement with Brain Volumes 

#### 3.2.1. ROI-Based Method

Inferences did not significantly differ for base models and fully adjusted models, so only fully adjusted models are presented. Higher social engagement was significantly associated with larger total brain volume and total GM volume, as well as larger temporal and occipital GM lobar volumes (Table 2), but not with total or lobar WM ROIs. Among the other ROIs evaluated, social engagement was only significantly associated with corpus callosum volume. There was no evidence of effect modification by age, education, race/ethnicity, or cardiovascular risk factors on relations of social engagement with ROI volumes. 

#### 3.2.2. Analysis to Discern Temporal Relationships

We evaluated whether changes in ROI volumes from the first to second MRI were associated with social engagement at the time of the second MRI. Information on changes in brain volumes in this cohort has been previously reported [18]. Changes in brain volumes over five years were not associated with social engagement at the end of the interval, except for temporal WM (*P* = 0.034) (Table 3). 

#### 3.2.3. Voxel-Based Method

**Figure 1:**
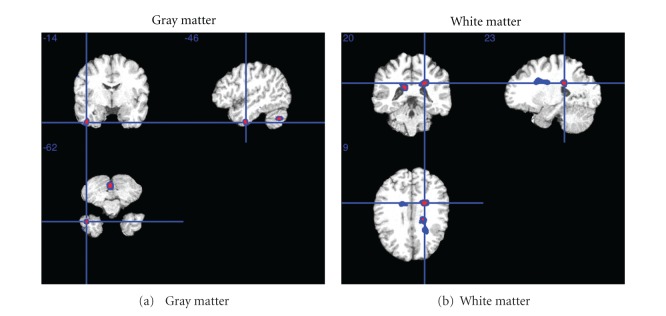
The highlighted zones indicate regions in which higher social activity was associated with larger brain volumes from voxel-based morphometry analysis. Only clusters of 100+ voxels shown. See Table 4 for cluster-specific statistics.

Clusters of voxels were identified in both GM and WM where social engagement was associated with larger voxel volume after adjustment for the aforementioned covariates (Table 4, Figure 1). There were twelve GM clusters of 100 or more voxels exceeding a statistical threshold of *t* = 3.11 (*P* < 0.001). The largest GM cluster was 4581 voxels (peak *t *= 4.23, *P* < 0.0001) and the second largest was 2501 voxels (peak *t *= 4.23, *P* < 0.0001). The permutation test-based *P* values for the largest two clusters were 0.05 and 0.14, respectively. Although social engagement was not associated with total or lobar WM ROIs, the VBM analysis identified six suprathreshold WM clusters of more than 100 contiguous voxels. The largest two WM clusters consisted of 3221 voxels (peak *t* = 3.95, *P* < 0.001) and 2592 voxels (peak *t* = 4.06, *P* < 0.0001), respectively. These clusters were localized to the interior regions near the cerebral fissure. Applying the cluster-based permutation test, the *P* value for the largest cluster was 0.10.

As the VBM analysis was not constrained by anatomical regions, there were a number of similarities as well as differences. Significant GM clusters were observed in the temporal lobe (Figure 1), the region found to have the strongest association with social engagement in the ROI analysis, but a number of clusters were observed in regions not identified by the ROI analysis, including clusters in the parietal lobe and cerebellum. Furthermore, there were no significant associations with lobar WM volumes in the ROI analysis, but large significant clusters were observed in WM in the VBM analysis. These were in the corpus callosum, which aligns with the ROI analysis.

## 4. Discussion

These findings provide some of the first published evidence that higher social engagement is associated with larger brain volumes as assessed by structural MRI using ROI- and voxel-based methods. In contrast, change in brain volumes over the five-year-period was not associated with social engagement. Therefore, these findings are consistent with the hypothesis that social engagement preserves brain tissue, and provide some evidence against the alternate hypothesis that persons with smaller or shrinking volumes become less socially engaged, although we cannot rule out the possibility that changes in social engagement over a longer period than five years may be associated with later volumes. The primary associations were with temporal and occipital lobar GM volumes, and likely as a result of this, with total GM and total brain volumes. There were no associations with lobar WM volumes. The findings support the brain reserve hypothesis by providing evidence that social engagement is associated with larger brain volumes in specific regions, which may in turn help to preserve cognitive function at older ages. 

There are a number of proposed biological mechanisms by which social engagement could affect cognitive function through changes in brain volume, especially in GM, the site of the neuronal cell bodies and a variety of connections between neural and glial tissues. Total brain volume loss [27], GM loss [28], neuronal shrinkage [29], and synaptic loss [30] are common consequences of aging. Neuronal and synaptic loss, as well as accelerated gross atrophy, are well-documented pathophysiologic correlates of early Alzheimer's disease [31]. Brain areas with larger volumes may be able to tolerate more loss caused by aging or disease before exhibiting declines in cognitive function because of a higher number of remaining healthy neurons and synapses [32]. Social engagement may lead to larger brain volumes through a decrease in neuronal death or shrinkage, neurogenesis in certain areas of the brain [12], increased dendritic spine growth or axonal rearrangement [33]. Other lines of research provide support for a link between social engagement and larger brain volumes, including associations between social engagement and other aspects of brain pathology or function and demonstrated neuroplastic increases in volume due to human behavior such as activity and learning. Furthermore, experiments with animals placed in enriched environments with increased opportunity for learning, activity, and interaction with other animals have demonstrated neurogenesis, synaptogensis, and reduced neuronal loss [11, 13, 34–36].

The localization of the social engagement-volume relationship should be interpreted with caution, but some initial conjectures can be made. The association with social engagement was strongest in GM in the right temporal lobe. Facial and verbal recognition, long-term memory, and personality features reside in the temporal lobe [37]. Loss of GM in this area occurs during aging [28]. GM was not significantly associated with social engagement in the frontal lobe, which displays the most loss during brain aging [38]. Furthermore, no significant associations were found for the hippocampus, a structure important to memory and cognition and vulnerable to aging [39], though some studies have shown preservation of hippocampal volumes with aging [40]. Research on enriched environments in animal models have found evidence of neurogenesis in the hippocampus, [14] and a study in humans showed larger hippocampi in socially engaged persons [41]. However, the hippocampus is a small structure and it is likely measured with less accuracy due to greater proportional error. The only WM structure found to be significantly associated with social engagement in the ROI-based analysis was the corpus callosum. There is some evidence that hemispheric asymmetry is a marker of reserve [42], and it is hypothesized that larger corpus callosum volume (which facilitates communication across hemispheres) may compensate for psychomotor slowing in later life [43]. Moreover, it is possible that preservation of lobar GM volumes could also preserve inter-hemispheric connections between those areas resulting in a larger corpus callosum.

Strengths of this study include the relatively large number of subjects with MRIs, the robustness of brain imaging analysis, the use of a rigorous measure of social engagement, the availability of longitudinal data, and the ability to control for important confounders. Conducting analyses using both ROIs and VBM gave us two separate but complimentary ways to examine the association between social engagement and tissue volume in specific areas of the brain [15]. The ROI analysis was informed by recognized anatomical structures while the VBM analysis did not rely on *a priori* structural boundaries but rather examined the entire brain in an unbiased region-by-region basis. 

One important limitation was the lack of a baseline social engagement measure, preventing us from examining the association between social engagement and change in brain structure or the association of change in social engagement with later brain structure. Another limitation is the unique nature of this cohort, which includes persons with past occupational lead exposure. Although we found no evidence that associations differed after control for lead dose or in comparing former lead workers to controls, the findings may not be generalizable to the general older adult population. The cohort is also racially and occupationally homogenous, and all male. Thus, we have no information on social engagement and the older female brain or differences across race/ethnic groups. However, homogeneity in occupation and socioeconomic status in this cohort may increase internal validity by lessening concerns of confounding by other factors linked to brain reserve and correlated with social engagement. Some potential confounders we were not able to adjust for include genes, IQ, stress response, personality, history of head injury, and MCI or prodromal dementia, all of which could be associated with both social engagement and brain structure. Finally, a limitation that could in part relate to the observed lack of an association between social engagement and WM is that we did remove white matter lesions from volumetric measures prior to analysis.

For more than a decade, recommendations have been made for older adults to stay socially engaged to keep their brains healthy based on evidence from epidemiologic studies of cognition and dementia. These studies have not addressed the “black box” regarding how social engagement may be related to the neuroanatomical substrate. Ours is an example, in a community-dwelling older adult population, of this necessary piece to the puzzle of why socially engaged persons are more cognitively intact at advanced ages.

## Figures and Tables

**Table 1:** Descriptive statistics.

	*N*	(%)	Social Engagement (Range −0.79, 1.09)	(SD)	Total brain volume (Range 880.6, 1449.3)	(SD)
All	348	(100%)	0.01	(0.33)	1137.4	(100.4)
Age						
48–59	82	(23.6%)	−0.07	(0.35)	1187.5	(94.6)
60–64	92	(26.4%)	0.04	(0.32)	1161.3	(90.6)
65–69	84	(24.1%)	0.02	(0.29)	1123.9	(81.8)
70–82	90	(25.9%)	0.07	(0.33)	1079.7	(100.4)
			*P* = 0.028		*P* < 0.001	
Race/ethnicity						
White/Non-Hispanic	315	(90.5%)	0.02	(0.32)	1141.6	(99.9)
All other	33	(9.6%)	−0.05	(0.40)	1097.1	(97.9)
			*P* = 0.25		*P* = 0.015	
Educational attainment						
<High school	25	(7.2%)	−0.05	(0.41)	1083.1	(74.7)
High school	90	(25.9%)	−0.05	(0.32)	1139.8	(97.4)
High school + trade school	167	(48.0%)	0.03	(0.30)	1135.8	(101.6)
College degree	56	(16.1%)	0.04	(0.34)	1171.6	(92.2)
Graduate degree	10	(2.9%)	0.28	(0.41)	1086.1	(137.9)
			*P* = 0.018		*P* = 0.002	
Hypertension						
Yes	180	(51.7%)	0.01	(0.32)	1116.6	(97.5)
No	168	(48.3%)	0.01	(0.33)	1159.6	(99.0)
			*P* = 0.97		*P* < 0.001	
Diabetes						
Yes	54	(15.5%)	−0.01	(0.29)	1098.8	(90.5)
No	294	(84.5%)	0.02	(0.34)	1144.4	(100.7)
			*P* = 0.59		*P* = 0.002	
Control status						
Population-based control	43	(12.4%)	0.12	(0.29)	1103.5	(100.5)
Former lead worker	305	(87.6%)	0.00	(0.33)	1142.1	(99.6)
			*P* = 0.018		*P* = 0.018	

All *P*-values from analysis of variance (ANOVA) tests.

**Table 2:** Adjusted associations between social engagement (independent variable) and ROI volumes (dependent variables).

ROI	Mean volume (cc)	Social engagement standardized coefficient	*P* value
**Total brain volume**	**1137.96**	**0.037**	**0.011**
**Total gray matter (GM)**	**534.14**	**0.072**	**0.007**
Total white matter (WM)	603.82	0.001	0.975
Gray matter lobes			
Frontal GM	134.85	0.037	0.273
**Temporal GM**	**96.58**	**0.083**	**0.009**
Parietal GM	65.40	0.068	0.081
**Occipital GM**	**45.56**	**0.076**	**0.048**
White matter lobes			
Frontal WM	201.47	0.008	0.761
Temporal WM	119.03	0.021	0.501
Parietal WM	106.08	0.026	0.456
Occipital WM	58.22	0.007	0.869
Smaller structures			
Cerebellum	119.63	−0.013	0.763
Medial structures	80.63	0.047	0.135
Cingulate gyrus	21.02	0.045	0.269
Insula	13.94	0.008	0.865
**Corpus callosum**	**11.89**	**0.127**	**0.004**
Internal capsule	9.85	0.026	0.520
Hippocampus	7.42	−0.004	0.923
Amygdala	2.43	0.037	0.453
Entorhinal cortex	2.37	−0.016	0.756

From models adjusted for age, education, intracranial volume, race/ethnicity, hypertension, diabetes, handedness, and control status.

Bold: *P* ≤ 0.05.

**Table 3:** Adjusted associations between change in ROI volumes (independent variable) and social engagement (dependent variable).

ROI	ΔROI standardized coefficient^1^	*P* value
Total brain volume	0.023	0.713
Total gray matter (GM)	0.062	0.299
Total white matter (WM)	−0.039	0.494
Gray matter lobes		
Frontal GM	0.032	0.599
Temporal GM	0.112	0.055
Parietal GM	0.092	0.133
Occipital GM	−0.011	0.849
White matter lobes		
Frontal WM	0.010	0.851
**Temporal WM**	−0.115	**0.034**
Parietal WM	−0.035	0.526
Occipital WM	0.046	0.416
Other structures (GM and WM)		
Cerebellum	0.022	0.695
Medial structures	0.005	0.937
Cingulate gyrus	−0.023	0.696
Insula	−0.014	0.803
Corpus callosum	−0.019	0.730
Internal capsule	0.067	0.244
Hippocampus	0.005	0.934
Amygdala	0.050	0.368
Entorhinal cortex	0.027	0.633

From models adjusted for age, education, intracranial volume, race/ethnicity, hypertension, diabetes, handedness, and control status.

^1^Social engagement at time of 2nd MRI was the dependent variable.

Bold: *P* ≤ 0.05.

**Table 4:** Cluster statistics from voxel-wise analysis.

*X*	*Y*	*Z*	Maximum *t*-statistic	Unadjusted *P* value	Cluster size	Cluster *P* value
Gray matter						

−42	50	−54	4.23	0.00002	4581	0.05
−3	51	−66	4.23	0.00001	2501	0.14
−65	−17	21	3.97	0.00004	1278	0.34
64	4	15	3.78	0.00009	1029	0.43
−46	−14	−63	4.16	0.00002	908	0.48
−56	−32	−64	4.08	0.00003	892	0.49
−65	17	−14	3.86	0.00007	511	0.68
−57	66	−25	3.9	0.00006	315	0.74
57	1	−46	3.73	0.00011	272	0.76
17	71	−31	3.54	0.00023	227	0.79
30	−37	32	3.47	0.00029	162	0.82
−50	14	−39	3.26	0.00062	119	0.82

White matter						

24	−21	12	3.95	0.00005	3221	0.10
−14	18	3	4.06	0.00003	2592	0.14
23	20	9	4.12	0.00002	1904	0.23
13	−47	−15	3.45	0.00031	797	0.44
35	45	−21	3.46	0.00030	198	0.77
27	41	−6	3.33	0.00048	102	0.83

Displays cluster centroid (*x*-, *y*-, and *z*- MNI coordinates), maximum *t*-statistic within the cluster, *P* value (unadjusted) of the maximum *t*-statistic, cluster size in number of contiguous voxels, and permutation test-based cluster *P* value. The cluster *P*-value compares the size of each suprathreshold cluster to the permutation distribution of the largest cluster, thereby accounting for multiple comparisons. Only results from clusters of 100+ voxels shown.

**Table 5:** Social engagement assessment.

Item: in the last week/month have you …	Response scale	Mean (std dev)
(i) been in touch with friends or relatives by phone or by letters?	W	2.6 (1.2)
(ii) gotten your hair [MEN cut WOMEN done] or dressed up to go out at least once?	M	2.5 (1.3)
(iii) done any unpaid volunteer work or community service?	M	0.9 (1.5)
(iv) been out to have lunch or dinner with someone?	M	2.8 (1.4)
(v) been to a meeting at a club, senior center, or organization in which you are active other than religious institution?	M	0.8 (1.2)
(vi) been out socially with friends or relatives, for example, to see a show, a party or holiday celebration, or some other social event?	M	1.2 (1.1)
(vii) gone shopping for food, clothes, or something else you needed?	M	2.8 (1.2)
(viii) done any indoor or outdoor recreational activity like bowling, working out, fishing, hiking, boating, swimming, golfing?	M	1.8 (2.0)

**Table 6:** The 8 social engagement items form one factor, of four, in the 20-item enacted function profile. Measurement properties of the enacted function profile follow.

Chi-square test of model fit	
Value	68.437
Degrees of freedom	46
*P* value	0.018
CFI/TLI	
Comparative fit index (CFI)	0.950
Tucker-lewis fit index (TLI)	0.946
RMSEA (Root mean square error of approximation)	
Estimate	0.035
WRMR (Weighted root mean square residual)	
Value	0.676

## Appendix

See Tables 5 and 6.
